# Nurse's Butter

**Published:** 1906-11-10

**Authors:** Harry Hems

**Affiliations:** Fair Park, Exeter


*To the Editor of* The Hospital.

### NURSE'S BUTTER.

*To the Editor of* The Hospital.

Sir,—In one of the several hospitals with which I have long been closely identified the question has arisen as to what amount of butter is a fair allowance for the nursing staff. At present—as for long past—the quantity per head is half-a-pound per week. A member of the managing board stoutly affirms this amount is not exceeded in any hospital in the world. This is a rather sweeping assertion! I have just come home across the Atlantic, and, being a bit of a "globe-trotter," should not like to give myself away by making such a statement. Still, the gentleman in question may have ground for his belief; hence information upon the subject from some of your readers will be valued.

I may add that, as one who has brought up a large family, I find my womenkind affirm the amount consumed per head in my own home is, on an average, considerably greater than half-a-pound a week.

Yours obediently,

Fair Park, Exeter, November 2, 1906. Harry Hems.

